# Supplementary material for: Interactions in psychosocial interventions in dementia care: A systematic review protocol
Source: PLoS One. 2026 May 6;21(5):e0348775. doi: 10.1371/journal.pone.0348775 (PMC13148684; doi:10.1371/journal.pone.0348775)
Supplement: S1 File — Completed PRISMA 2020 checklist for this systematic review protocol. (DOCX) [file pone.0348775.s001.docx]

| Search # | Subject Headings (MH) and Key Words | Articles Revealed |
| --- | --- | --- |
| S1 | (MH "Dementia") OR (MH "Alzheimer Disease") OR (MH "Lewy Body Disease") OR (MH “primary progressive aphasia”) OR “Frontotemporal dementia” OR (MH “MCI”) OR (MH “cognitive impairment”) OR (MH “neurocognitive disorder”) OR “dementia” OR “Alzheimer Disease” OR “Alzheimer” OR “Lewy” OR "Vascular dementia” OR “alcohol related dementia” OR “Parkinson disease dementia” OR “Lewy Body Disease” OR “MCI” OR “cognitive impairment” OR “neuro-cognitive” |  |
| S2 | (MH "Cognitive Therapy") OR (MH "Behavior Therapy") OR (MH "Psychotherapy") OR (MH "Reality Therapy") OR (MH "Exercise") OR (MH "Massage") OR (MH "Aromatherapy") OR (MH "Animal Assisted Therapy") OR (MH "Art Therapy") OR (MH "Music Therapy") OR (MH "Occupational Therapy") OR (MH "Play Therapy") OR (MH "Recreational Therapy") OR (MH "meditation") OR (MH "mindfulness") OR "Behavioral Disciplines and Activities" OR "Non pharmacological" OR "non drug" OR “behavioral” OR "Behavior Therapy" OR “psychosocial” OR "Psychodynamic therapy" OR “Psychotherapy” OR "Psychological treatment" OR "Cognitive Stimulation Therapy" OR "Cognitive Therapy" OR "cognitive training" OR "cognitive rehabilitation" OR "Reality Orientation" OR “Reminiscence” OR “Reminiscences” OR “RT” OR “autobiography” OR "life review" OR “storytelling” OR "oral history" OR "life history" OR "validation therapy” OR "Reality Therapy" OR "dementia care mapping" OR "Montessori based activities" OR "ability focused" OR “Exercise” OR “Physical” OR "muscle relaxation" OR “touch” OR "touch therapy" OR “Massage” OR "Multi component therapy" OR “Aromatherapy” OR "Animal Assisted Therapy" OR "doll therapy" OR "robot therapy" OR "Horticultural Therapy" OR "Art Therapy" OR “painting” OR "Spaced retrieval" OR "Music Therapy" OR "light therapy" OR "Occupational Therapy" OR "Play Therapy" OR "Recreation Therapy" OR “snoezelen” OR "multimodality therapy" OR “sensory” OR "sensory stimulation" OR "emotion oriented" OR "therapeutic conversation" OR “meditation” OR “mindfulness” OR "Special care" OR “speech therapy” OR “language therapy” OR “conversation therapy” OR “communication therapy” |  |
| S3 | ((MH "interact") OR (MH “conversation”) OR (MH “language”) OR (MH “speech”) OR (MH “gesture”) OR (MH “communication”) OR (MH “nonverbal”) OR “interact” OR “conversation” OR “language” OR “speech” OR “gesture” OR “communication” OR “nonverbal” OR “talk” OR “word” OR “speak” OR “express” OR “engage” OR “vocalization” OR “eye gaze” OR “eye contact” OR “attention” OR “smile” OR “laughter” OR “behavior” OR “react” OR “response” OR “physical” OR “emotion”) |  |
| S4 | S1 AND S2 AND S3 |  |

Studies were excluded if they 1) were publications in languages other than English; 2) incomplete studies such as study protocols or ongoing studies; 3) did not have sufficient information about the measurement of the outcome of interest

Databases to search: PubMed, [CINAHL](https://www.sciencedirect.com/topics/nursing-and-health-professions/cinahl), [PsycINFO](https://www.sciencedirect.com/topics/nursing-and-health-professions/psycinfo), [Embase](https://www.sciencedirect.com/topics/nursing-and-health-professions/embase), and the [Cochrane Library](https://www.sciencedirect.com/topics/nursing-and-health-professions/cochrane-library)

((MH "Dementia") OR (MH "Alzheimer Disease") OR (MH "Lewy Body Disease") OR (MH “primary progressive aphasia”) OR (MH “MCI”) OR (MH “cognitive impairment”) OR (MH “neurocognitive disorder”) OR “dementia” OR “Alzheimer Disease” OR “Alzheimer” OR “Lewy” OR "Vascular dementia” OR “alcohol related dementia” OR “Parkinson disease dementia” OR “Lewy Body Disease” OR “MCI” OR “cognitive impairment” OR “neuro-cognitive”) AND ((MH "Cognitive Therapy") OR (MH "Behavior Therapy") OR (MH "Psychotherapy") OR (MH "Reality Therapy") OR (MH "Exercise") OR (MH "Massage") OR (MH "Aromatherapy") OR (MH "Animal Assisted Therapy") OR (MH "Art Therapy") OR (MH "Music Therapy") OR (MH "Occupational Therapy") OR (MH "Play Therapy") OR (MH "Recreational Therapy") OR (MH "meditation") OR (MH "mindfulness") OR "Behavioral Disciplines and Activities" OR "Non pharmacological" OR "non drug" OR “behavioral” OR "Behavior Therapy" OR “psychosocial” OR "Psychodynamic therapy" OR “Psychotherapy” OR "Psychological treatment" OR "Cognitive Stimulation Therapy" OR "Cognitive Therapy" OR "cognitive training" OR "cognitive rehabilitation" OR "Reality Orientation" OR “Reminiscence” OR “Reminiscences” OR “RT” OR “autobiography” OR "life review" OR “storytelling” OR "oral history" OR "life history" OR "validation therapy” OR "Reality Therapy" OR "dementia care mapping" OR "Montessori based activities" OR "ability focused" OR “Exercise” OR “Physical” OR "muscle relaxation" OR “touch” OR "touch therapy" OR “Massage” OR "Multi component therapy" OR “Aromatherapy” OR "Animal Assisted Therapy" OR "doll therapy" OR "robot therapy" OR "Horticultural Therapy" OR "Art Therapy" OR “painting” OR "Spaced retrieval" OR "Music Therapy" OR "light therapy" OR "Occupational Therapy" OR "Play Therapy" OR "Recreation Therapy" OR “snoezelen” OR "multimodality therapy" OR “sensory” OR "sensory stimulation" OR "emotion oriented" OR "therapeutic conversation" OR “meditation” OR “mindfulness” OR "Special care" OR “speech therapy” OR “language therapy” OR “conversation therapy” OR “communication therapy”) AND ((MH "interact") OR (MH “conversation”) OR (MH “language”) OR (MH “speech”) OR (MH “gesture”) OR (MH “communication”) OR (MH “nonverbal”) OR “interact” OR “conversation” OR “language” OR “speech” OR “gesture” OR “communication” OR “nonverbal” OR “talk” OR “word” OR “speak” OR “express” OR “engage” OR “vocalization” OR “eye gaze” OR “eye contact” OR “attention” OR “smile” OR “laughter” OR “behavior” OR “react” OR “response” OR “physical” OR “emotion”)
