# Supplementary material for: Interactions in psychosocial interventions in dementia care: A systematic review protocol
Source: PLoS One. 2026 May 6;21(5):e0348775. doi: 10.1371/journal.pone.0348775 (PMC13148684; doi:10.1371/journal.pone.0348775)
Supplement: S2 File — Full search strategy for each database (PubMed, PsycINFO, MedLine, CINAHL, Cochrane Library). (DOCX) [file pone.0348775.s002.docx]

**PRISMA-P (Preferred Reporting Items for Systematic review and Meta-Analysis Protocols) 2015 checklist: recommended items to address in a systematic review protocol***

| Section and topic | Item No | Checklist item |
| --- | --- | --- |
| ADMINISTRATIVE INFORMATION | | |
| Title: |  | Interactions in psychosocial interventions in dementia care: A systematic review protocol |
| Identification | 1a | Interactions in psychosocial interventions in dementia care: A systematic review protocol |
| Update | 1b | This protocol is for a new systematic review, not an update of a previously published or registered systematic review. |
| Registration | 2 | The study protocol has been registered at the Campbell Systematic Reviews with the registration number cl2.20250120. |
| Authors: |  | Ivy Meihua Su^1^, Keyu Li^1^, Winsy Wing Sze Wong^1^* |
| Contact | 3a | *Corresponding author: Winsy Wing Sze Wong  [winsyws.wong@polyu.edu.hk](mailto:winsyws.wong@polyu.edu.hk)  Physical mailing address: Department of Language Science and Technology, Faculty of Humanities, The Hong Kong Polytechnic University, 11 Yuk Choi Rd, Hung Hom, Kowloon, Hong Kong SAR, China  Author Affiliations  1Department of Language Science and Technology, Faculty of Humanities, The Hong Kong Polytechnic University, 11 Yuk Choi Rd, Hung Hom, Hong Kong SAR, China  Email: Ivy Meihua Su [Ivy-meihua.su@connect.polyu.hk](mailto:Ivy-meihua.su@connect.polyu.hk) - Keyu Li [24107978d@connect.polyu.hk](mailto:24107978d@connect.polyu.hk) - [winsyws.wong@polyu.edu.hk](mailto:winsyws.wong@polyu.edu.hk) |
| Contributions | 3b | Winsy Wing Sze Wong is the guarantor. Ivy Meihua Su and Keyu Li drafted the protocol. All authors contributed to the development of the eligibility criteria, data extraction plan, and screening strategy. Keyu Li conducted the initial search. Winsy Wing Sze Wong conceptualized the review and supervised the protocol development. All authors critically revised and approved the final version of the protocol. |
| Amendments | 4 | This protocol is the initial version. Any future amendments will be documented in a dedicated “Protocol Amendments” section, including: (1) date of amendment, (2) detailed description of changes, (3) rationale for changes. |
| Support: |  |  |
| Sources | 5a | This protocol is supported by the Health and Medical Research Fund of the Health Bureau, The Government of the Hong Kong Special Administrative Region (Ref: 22231471) and the General Research Fund of the Research Grants Council of the Hong Kong Special Administrative Region (Ref: 15602024). |
| Sponsor | 5b | The sponsors are the Health Bureau (HMRF) and the Research Grants Council (GRF) of the Hong Kong Special Administrative Region. |
| Role of sponsor or funder | 5c | The funders had no role in study design, data collection and analysis, decision to publish, or preparation of the manuscript. |
| INTRODUCTION | | |
| Rationale | 6 | Dementia is a progressive neurocognitive disorder that significantly impairs cognitive and functional abilities, affecting over 55 million people worldwide. While pharmacological treatments for dementia offer only marginal benefits and are often associated with adverse effects, psychosocial interventions have emerged as preferable alternatives. Psychosocial interventions, which include therapies such as music, reminiscence, animal-assisted, and cognitive stimulation, focus on psychosocial, environmental, and behavioral strategies to enhance well-being, preserve function, and reduce distress in people living with dementia.  A key component of psychosocial interventions is the interactional behavior of people living with dementia, encompassing verbal, nonverbal, and multimodal exchanges with caregivers, peers, stimuli, and the environment. These interactions are believed to underpin engagement and participation in psychosocial interventions, which in turn may influence therapeutic outcomes such as cognitive function, psychological well-being, and quality of life. Existing research has demonstrated the benefits of psychosocial interventions on various outcomes, but the mechanisms by which interactions contribute to these effects remain underexplored.  Previous studies have developed a range of tools and frameworks to measure interactions in dementia care, often focusing on communication between people living with dementia and caregivers or healthcare professionals. However, these measures vary widely in their focus (e.g., verbal vs. nonverbal), methodological approach (quantitative vs. qualitative), and the extent to which they capture the mutual and dynamic nature of interactions in structured therapeutic settings. Moreover, there is a lack of comprehensive synthesis regarding how interactions are measured across different psychosocial interventions and how these measures relate to treatment outcomes.  Given the diversity of psychosocial interventions and the centrality of interaction in their delivery, a systematic review is needed to synthesize existing methods and perspectives on measuring interactions in dementia therapies. Such a review will help clarify the current landscape of interaction measurement, identify gaps in the literature, and inform the development of more robust and comprehensive assessment tools. Ultimately, this work aims to advance understanding of how interactions within psychosocial interventions contribute to improved outcomes for people living with dementia. |
| Objectives | 7 | This systematic review addresses four research questions using the PICO framework:  - **Participants**: People living with dementia of any type (Alzheimer’s Disease, dementia with Lewy bodies, vascular dementia, etc.) and severity; caregivers/facilitators involved in people living with dementia’s psychosocial interventions.  - **Interventions**: Psychosocial interventions for people living with dementia (music therapy, robot therapy, animal therapy, cognitive stimulation therapy, reminiscence therapy, etc.), with a focus on interaction processes.  - **Comparators**: Not applicable (review synthesizes measurement methods, not intervention comparisons).  - **Outcomes**:  1. What are the nature/characteristics of studies measuring people living with dementia’s interactions in psychosocial interventions (e.g., psychosocial intervention type, participant demographics)?  2. Which aspects of interactions (verbal vs. nonverbal) are measured in psychosocial interventions?  3. How are interactions measured in different psychosocial intervention settings (e.g., tools, parameters, qualitative/quantitative approaches, coding systems)?  4. Do studies investigate the relationship between interaction measures and psychosocial intervention outcomes (cognitive, behavioral, QoL, mood, engagement)? If so, further investigation will be carried out to describe the relationship between measures and outcomes. |
| METHODS | | |
| Eligibility criteria | 8 | Studies will be selected according to the criteria outlined below.  Study designs:   - **Participants (P):**   - People living with dementia of any type (e.g., Alzheimer’s Disease, dementia with Lewy bodies, vascular dementia, alcohol-related dementia, Parkinson’s disease dementia, primary progressive aphasia), at any stage of severity (mild, moderate, or severe).   - Studies involving caregivers and facilitators in relation to the targeted population are eligible.   - Studies involving only the general elderly population or a mixed population (dementia and non-dementia) where people living with dementia are less than 80% will be excluded. - **Interventions (I):**   Psychosocial interventions for people living with dementia, such as music therapy, robot therapy, animal therapy, bright light therapy, cognitive stimulation therapy, reminiscence therapy, and other structured activities involving interaction (person-environment, person-caregiver, person-stimulus, or group-based).   - - Studies employing pharmacological approaches or not measuring the interactional process in psychosocial interventions will be excluded. - **Comparators (C):**   - Studies will be included regardless of the presence or absence of a comparator group. Where present, comparators may include different psychosocial interventions, usual care, or alternative interventions. - **Outcomes (O):**   - Primary outcome: Measurement of interaction processes (e.g., observation scales, coding systems, quantitative or qualitative analysis) among people living with dementia and their environment/caregivers/peers/stimuli/facilitators during psychosocial interventions.   - Secondary outcomes: Any reported association between interaction measures and dementia care outcomes (e.g., cognitive, behavioral, psychological, quality of life, mood, engagement). - **Study Design:**   - Eligible: Empirical studies (randomized controlled trials, non-randomized controlled trials, observational studies, pre- and post-studies, qualitative studies), and psychometric studies of scales.   - Excluded: Review articles, scoping reviews, systematic reviews, and theoretical papers. - **Setting:**   - Any setting where psychosocial interventions for people living with dementia are delivered (e.g., care homes, community centers, hospitals, outpatient clinics, or home-based settings). - **Time Frame:**   - Studies published up to December 31, 2025.   **Report Characteristics**   - **Years Considered:**   - All years up to December 31, 2025. - **Language:**   - No restrictions on publication language will be imposed. - **Publication Status:**   - Only published studies will be included. Grey literature (e.g., dissertations, conference abstracts) will be considered if they meet all other eligibility criteria. |
| Information sources | 9 | **Electronic databases:** The following bibliographic databases will be searched: PubMed, PsycINFO (ProQuest), MedLine (EbscoHost), CINAHL (EbscoHost), and the Cochrane Library. **Grey literature:** A supplementary search for grey literature will be conducted using **ProQuest Dissertations and Theses Global** and **Google Scholar** (screening the first 200 results). Additionally, proceedings of major conferences (e.g., Alzheimer’s Association International Conference) from the last three years will be screened. **Other sources:** Backward citation tracking will be performed by manually screening the reference lists of all included studies and relevant systematic reviews. All sources will be searched for studies published up to December 31, 2025. |
| Search strategy | 10 | **Database search:** Search strategies will be developed for each electronic database using a combination of controlled vocabulary (MeSH) and free-text terms related to three key concepts: population (people living with dementia), intervention (psychosocial/nonpharmacological), and outcome (interaction). A draft PubMed search strategy is included in Appendix 1. **Grey literature search:** Simplified combinations of key search terms (e.g., “dementia” AND “interaction” AND “psychosocial”) will be used for Google Scholar and ProQuest Dissertations and Theses. No language restrictions will be imposed; non-English studies will undergo initial machine translation for screening. Where necessary, study authors may be contacted for clarification or to obtain further data.  The search will cover all years up to December 31, 2025. The search will be updated toward the end of the review to ensure currency. The search strategy will be validated by checking whether it retrieves a high proportion of eligible studies, including those identified through other means but not indexed in the primary databases. |
| Study records: |  |  |
| Data management | 11a | Record management: Search results from all databases will be imported into Covidence systematic review software to remove duplicates (identified by title, author, publication year, and study characteristics). Citations will be organized using EndNote reference management software.  Data storage: Extracted data will be stored in a password-protected Microsoft Excel spreadsheet, with encrypted backups saved on the institutional server of The Hong Kong Polytechnic University. All records (screened, excluded, included) will be tracked in Covidence, with reasons for exclusion documented at both title/abstract and full-text stages.  Duplicate study handling: Multiple reports of the same study will be identified via author names, study design, sample size, and intervention details; data from all reports will be merged to ensure completeness, and the primary report (most comprehensive) will be labeled as the main reference. |
| Selection process | 11b | Study selection will involve two independent reviewers (Ivy Meihua Su and Keyu Li) in two phases:  1. Title/abstract screening: Reviewers will independently assess citations against eligibility criteria; citations marked as “eligible” or “uncertain” will proceed to full-text screening.  2. Full-text screening: Reviewers will independently evaluate full texts of preliminarily eligible studies; a third reviewer (Winsy Wing Sze Wong) will resolve unresolved disagreements.  Inter-rater reliability: A 10% random sample of citations (both included and excluded) will be cross-checked by the two reviewers to ensure ≥90% inter-rater reliability. If reliability is not met, reviewers will undergo calibration (reviewing 5 additional studies together) before resuming screening.  Exclusion documentation: Reasons for full-text exclusion will be recorded in a standardized form (e.g., “study does not measure interaction processes”). |
| Data collection process | 11c | **Data extraction form**: A standardized extraction form will be developed via pilot testing on 5 randomly selected eligible studies; the form will be revised to address ambiguities identified during piloting.  **Extraction process**: Two independent reviewers (Ivy Meihua Su and Keyu Li) will extract data in duplicate using the finalized Excel form. Reviewers will not be blinded to study authors, journals, or institutions.  **Missing data**: For unreported data (e.g., dementia severity, interaction measurement parameters), reviewers will contact original study authors via email (up to 3 attempts at 2-week intervals). If no response is received, missing data will be recorded as “not reported” and noted in the synthesis.  **Quality control**: Extracted data will be cross-checked by the two reviewers; disagreements will be resolved via discussion or adjudication by the third reviewer (Winsy Wing Sze Wong). |
| Data items | 12 | The following variables will be extracted, with definitions provided:  1. Study characteristics: Author, publication year, country, study design (e.g., RCT, qualitative ethnography), setting (e.g., nursing home, community center), funding source (if reported).  2. Participant characteristics: Sample size (people living with dementia, caregivers), dementia type (e.g., Alzheimer’s Disease, based on study definition), dementia severity (mild/moderate/severe, per study criteria), demographics (mean age, gender distribution, education level).  3. Intervention details: psychosocial interventions type (e.g., robot therapy), session duration (minutes/session), total intervention duration (weeks), frequency (sessions/week), facilitator type (e.g., occupational therapist), specific interaction-focused activities (e.g., “people living with dementia interact with PARO robot via touch”).  4. Interaction measurement items:  - Aspects: Verbal (e.g., speech frequency, turn-taking, content relevance) vs. nonverbal (e.g., eye contact, facial expressions, physical touch).  - Tool/method: Name of scale (e.g., VNVIS-CR), observational coding system, or qualitative framework (e.g., symbolic interactionism).  - Parameters: Metrics measured (e.g., “verbal utterances per minute,” “duration of eye contact”).  - Nature: Quantitative (e.g., counts, durations), qualitative (e.g., thematic analysis), or mixed methods.  5. Outcome items: psychosocial interventions outcomes measured (cognitive: e.g., MMSE; behavioral: e.g., Cohen-Mansfield Agitation Inventory; QoL: e.g., QOL-AD), outcome measurement tools, and reported interaction-outcome relationships (e.g., correlation coefficients, qualitative themes).  Pre-planned simplifications: If studies report multiple parameters for the same interaction aspect (e.g., “verbal frequency” and “verbal content”), the most frequently analyzed parameter (per study) will be extracted; if no clear priority, all parameters will be synthesized separately. |
| Outcomes and prioritization | 13 | **Main outcomes** (priority rationale: address core gap of fragmented interaction measurement frameworks):  1. Methods of measuring interactions in psychosocial interventions (tools, parameters, qualitative/quantitative approaches, coding systems).  2. Aspects of interactions measured (verbal vs. nonverbal).  **Additional outcomes** (priority rationale: provide context for measurement methods and practical relevance):  1. Nature/characteristics of studies measuring people living with dementia’s interactions (e.g., psychosocial intervention type, geographical distribution).  2. Relationship between interaction measures and psychosocial intervention outcomes (cognitive, behavioral, QoL, mood, engagement).  **Rationale for prioritization**: Establishing standardized approaches to measuring interactions is essential for advancing the rigor and comparability of psychosocial intervention research; thus, methods/aspects of measurement are prioritized over contextual or outcome-related data, which support but do not drive the review’s core goal. |
| Risk of bias in individual studies | 14 | **Quantitative studies:**The methodological quality will be assessed using the Joanna Briggs Institute (JBI) Critical Appraisal Tools. Specifically, the *JBI Checklist for Randomized Controlled Trials* will be used for RCTs, the *JBI Checklist for Quasi-Experimental Studies* for non-randomized experimental studies, and the *JBI Checklist for Analytical Cross-Sectional Studies* for observational designs. These tools assess key domains such as randomization, blinding, and statistical analysis.  **Psychometric studies:** For studies focusing specifically on the psychometric properties of interaction scales, the COSMIN Risk of Bias checklist will be utilized to evaluate validity and reliability.  **Qualitative studies**: Assessed using the *CASP Qualitative Checklist* (2018) at the study level, evaluating 10 domains: research question clarity, methodological appropriateness, participant recruitment rationale, data collection rigor, data analysis transparency, reflexivity (researcher bias acknowledgment), credibility (member checking, if done), transferability (contextual detail), dependability (audit trail), and confirmability (avoidance of researcher bias).  **Assessment process**: Two independent reviewers (Ivy Meihua Su and Keyu Li) will conduct bias assessments; disagreements will be resolved via discussion or third reviewer adjudication.  **Use in synthesis**: High-risk studies will be included in the narrative synthesis but clearly labeled; sensitivity analysis (if feasible) will explore whether excluding high-risk studies alters conclusions about interaction-outcome relationships. |
| Data synthesis | 15a | Study data will be quantitatively synthesised if the included studies are sufficiently homogeneous in terms of participants, interventions, outcome measures, and study design. Specifically, quantitative synthesis (such as meta-analysis) will be considered if:   - Studies report on similar types of psychosocial interventions for people living with dementia. - The same or comparable measures of interaction (e.g., frequency, duration, or standardized scales of interaction) are used across studies. - Outcomes related to the association between interaction measures and dementia care outcomes (such as cognitive function, behavioral symptoms, psychological well-being, or quality of life) are reported in a way that allows for pooling of effect sizes (e.g., means and standard deviations, odds ratios, correlation coefficients). - Study designs are sufficiently similar (e.g., randomized controlled trials, controlled before-and-after studies, or observational studies with comparable methodologies).   If these criteria are met, and there is an adequate number of studies (typically at least two or more) reporting compatible data, a quantitative synthesis will be conducted. Heterogeneity will be assessed using statistical measures (such as the I² statistic), and if substantial heterogeneity is detected, a random-effects model will be used or, if appropriate, a narrative synthesis will be provided instead.  If the data are too heterogeneous in terms of interventions, outcome measures, or study designs, or if insufficient quantitative data are available, a narrative synthesis will be undertaken. |
|  | 15b | If the included studies are appropriate for quantitative synthesis, summary measures will be selected based on the type of outcome data reported. For continuous outcomes (such as frequency or duration of interactions, or scores on interaction scales), mean differences or standardized mean differences (SMD) with 95% confidence intervals will be calculated. For dichotomous outcomes (such as presence or absence of a specific interaction behavior), risk ratios (RR) or odds ratios (OR) with 95% confidence intervals will be used. For studies reporting associations between interaction measures and outcomes (e.g., cognitive function, quality of life), correlation coefficients or regression estimates will be extracted and, where possible, pooled.  Data from individual studies will be extracted into a standardized spreadsheet. Where necessary, authors will be contacted for missing or unclear data. If studies report data in different formats, appropriate statistical conversions will be applied to ensure comparability (e.g., converting medians and interquartile ranges to means and standard deviations).  Data will be combined using meta-analysis if at least two studies report sufficiently similar outcomes and measures. A random-effects model will be used to account for potential heterogeneity between studies. Statistical heterogeneity will be assessed using the I² statistic, with values of 25%, 50%, and 75% representing low, moderate, and high heterogeneity, respectively. Where appropriate, additional measures such as Kendall’s τ will be used to explore consistency across studies. If substantial heterogeneity is detected, subgroup analyses or sensitivity analyses will be conducted to explore possible sources (e.g., differences in intervention type, participant characteristics, or study design).  If quantitative synthesis is not appropriate due to high heterogeneity or insufficient data, results will be presented using narrative synthesis. |
|  | 15c | Subgroup analysis will be conducted narratively if data permits (e.g., comparing verbal vs. nonverbal tools). Statistical sensitivity analysis and meta-regression are not planned due to the anticipated heterogeneity of study designs. |
|  | 15d | If quantitative synthesis is not appropriate due to substantial heterogeneity in study designs, interventions, outcome measures, or insufficient quantitative data, a narrative synthesis will be conducted. The narrative synthesis will systematically summarize and describe the key characteristics and findings of the included studies. This will include:   - A structured summary of study characteristics, including participants, intervention types, settings, and outcome measures. - A thematic grouping of studies according to the type of psychosocial intervention, type of interaction measured, or other relevant features. - A descriptive account of the main findings related to the measurement of interactions and their association with dementia care outcomes. - Identification of patterns, similarities, and differences across studies, as well as any notable gaps in the evidence. - Where possible, tables and figures will be used to organize and present the data clearly.   The narrative synthesis will follow the Synthesis Without Meta-analysis (SWiM) reporting guidelines to ensure transparency and rigor in reporting the review findings. |
| Meta-bias(es) | 16 | Due to the qualitative and descriptive nature of the review objectives, mata-analysis including funnel plots and statistical tests for publication bias are not applicable. |
| Confidence in cumulative evidence | 17 | Not applicable. As this review focuses on measuring properties and methodological scope rather than intervention efficacy, assessment of the overall strength and certainty of the body of evidence for each main outcome is not appropriate. |

*** It is strongly recommended that this checklist be read in conjunction with the PRISMA-P Explanation and Elaboration (cite when available) for important clarification on the items. Amendments to a review protocol should be tracked and dated. The copyright for PRISMA-P (including checklist) is held by the PRISMA-P Group and is distributed under a Creative Commons Attribution Licence 4.0.**

*From: Shamseer L, Moher D, Clarke M, Ghersi D, Liberati A, Petticrew M, Shekelle P, Stewart L, PRISMA-P Group. Preferred reporting items for systematic review and meta-analysis protocols (PRISMA-P) 2015: elaboration and explanation. BMJ. 2015 Jan 2;349(jan02 1):g7647.*

**Appendix 1**

**Draft PubMed search strategy**

1. ("Dementia"[Mesh] OR "Alzheimer Disease"[Mesh] OR "Lewy Body Disease"[Mesh] OR "Primary Progressive Aphasia"[Mesh] OR “Frontotemporal dementia” OR "Mild Cognitive Impairment"[Mesh] OR "Cognitive Dysfunction"[Mesh] OR "Neurocognitive Disorders"[Mesh] OR dementia OR "Alzheimer Disease" OR Alzheimer OR Lewy OR "Vascular dementia" OR "alcohol related dementia" OR "Parkinson disease dementia" OR "Lewy Body Disease" OR MCI OR "cognitive impairment" OR neuro-cognitive)
2. ("Cognitive Therapy"[Mesh] OR "Behavior Therapy"[Mesh] OR "Psychotherapy"[Mesh] OR "Reality Therapy"[Mesh] OR "Exercise"[Mesh] OR "Massage"[Mesh] OR "Aromatherapy"[Mesh] OR "Animal Assisted Therapy"[Mesh] OR "Art Therapy"[Mesh] OR "Music Therapy"[Mesh] OR "Occupational Therapy"[Mesh] OR "Play Therapy"[Mesh] OR "Recreational Therapy"[Mesh] OR meditation OR mindfulness OR "Behavioral Disciplines and Activities" OR "Non pharmacological" OR "non drug" OR behavioral OR "Behavior Therapy" OR psychosocial OR "Psychodynamic therapy" OR "Psychological treatment" OR "Cognitive Stimulation Therapy" OR "cognitive training" OR "cognitive rehabilitation" OR "Reality Orientation" OR Reminiscence OR "life review" OR storytelling OR "oral history" OR "life history" OR "validation therapy" OR "dementia care mapping" OR "Montessori based activities" OR "ability focused" OR "muscle relaxation" OR touch OR "touch therapy" OR "Multi component therapy" OR "doll therapy" OR "robot therapy" OR "Horticultural Therapy" OR painting OR "Spaced retrieval" OR "light therapy" OR snoezelen OR "multimodality therapy" OR sensory OR "sensory stimulation" OR "emotion oriented" OR "therapeutic conversation" OR "Special care" OR "speech therapy" OR "language therapy" OR "conversation therapy" OR "communication therapy")
3. (interact OR "interact"[Mesh] OR conversation OR "conversation"[Mesh] OR language OR "language"[Mesh] OR speech OR "speech"[Mesh] OR gesture OR "gesture"[Mesh] OR communication OR "communication"[Mesh] OR nonverbal OR "nonverbal"[Mesh] OR talk OR word OR speak OR express OR engage OR vocalization OR "eye gaze" OR "eye contact" OR attention OR smile OR laughter OR behavior OR react OR response OR physical OR emotion)
4. 1 AND 2 AND 3

**Limits applied:** publication date up to December 31, 2025.
