# Supplementary material for: Interactions in psychosocial interventions in dementia care: A systematic review protocol
Source: PLoS One. 2026 May 6;21(5):e0348775. doi: 10.1371/journal.pone.0348775 (PMC13148684; doi:10.1371/journal.pone.0348775)
Supplement: Attachment — Submitted filename: response letter.docx [file pone.0348775.s003.docx]

November 26, 2025

Dear Editor,

**Response letter to the Submission of Revised Manuscript PONE-D-25-55362 – "Interactions in dementia therapies: A systematic review protocol"**

We sincerely appreciate your decision of October 17, 2025, and the constructive comments from the editorial team, which have been invaluable in strengthening the methodological rigor of our systematic review protocol.

This study aims to map and synthesize evidence on the nature and impact of interactions within multidisciplinary dementia therapies, addressing a critical gap in the literature by focusing on the process elements of care.

In revising the manuscript, we have paid particular attention to the core concern regarding language restrictions in the search strategy. The specific changes implemented are as follows:

Comment from the editor:

In the case of your submission, we note that language restrictions are imposed on the literature searches without adequate justification. There is increasing recognition that exclusion of studies published in other languages can increase the risk of bias in systematic reviews. We feel that any restrictions should be justified based on scientific reasons rather than resource constraints. As a result, we have concerns about the comprehensiveness of the review and the validity of the conclusions.

As a result of these concerns, we cannot consider the manuscript for publication at PLOS ONE at this time. However, if you are able to address the concerns discussed above, we may be able to consider a revised version of the manuscript. This would however require a substantial amount of work and thus, would be considered as a new submission. If you are able to revise the manuscript to respond to the concerns noted above, please refer to the original submission in the covering letter and accompany the submission by a rebuttal letter that responds to the comments made on the original manuscript.

Please find our response below:

**Primary Concern: Justification for Language Restriction**

We fully acknowledge that the original submission imposed language restrictions without sufficient scientific justification, which could have introduced selection bias. In response, we have taken the following steps:

1. **Removed All Language Restrictions:** We have removed the language restriction in the exclusion criteria (page 12, line 17) and revised the “Eligibility criteria” section (page 12, line 3) to explicitly state: “No restrictions on publication language will be imposed.”
2. **Implemented a Rigorous Translation Protocol:** To ensure the accurate inclusion and data extraction of non-English studies, we have established a robust, multi-step process detailed in the methodology:
   - Studies in languages not fluently spoken by the author team will undergo initial machine translation (e.g., Google Translate) for screening (page 12, line 4).
   - For all studies included in the final review, key data (e.g., outcomes, methodology, results) will be translated by a fluent academic colleague or a professional translation service to guarantee accuracy (page 12, lines 5–7).
   - We will contact the original authors, where feasible, to verify critical data interpretations and confirm their accuracy. (page 12, lines 8-9).
3. **Ensured PRISMA-P Compliance:** The PRISMA-P checklist has been updated accordingly in **Item 10 (Search Strategy) in lines 1-3, and the last line of Appendix 1 (Limits Applied)** to reflect the removal of language restrictions and the outlined translation methodology.
4. **Revised the supporting information:** The language restriction has been removed in S1 Appendix. Search Terms (line 5).

We are confident that these revisions have thoroughly addressed the editorial concern. The updated protocol now enhances the comprehensiveness of the review, minimizes potential bias, and aligns with best practices in systematic reviewing.

Thank you again for the comments. We are now submitting the revised version, as per your guidelines, and hope it now meets the criteria for publication in *PLOS ONE*.

Sincerely,
Winsy Wing Sze Wong, Ph.D.
Corresponding Author
Department of Language Science and Technology
The Hong Kong Polytechnic University
