# Supplementary material for: Interactions in psychosocial interventions in dementia care: A systematic review protocol
Source: PLoS One. 2026 May 6;21(5):e0348775. doi: 10.1371/journal.pone.0348775 (PMC13148684; doi:10.1371/journal.pone.0348775)
Supplement: Attachment — Submitted filename: 3rd response letter.docx [file pone.0348775.s004.docx]

March 18, 2026

Response Letter

**Manuscript ID**: PONE-D-25-60484R1

**Title**: Interactions in psychosocial interventions in dementia therapies: A systematic review protocol

Dear Ms. Trisha Mae Tañedo Perez,

Thank you for your detailed feedback and guidance on our manuscript (PONE-D-25-60484R1). We have carefully addressed all the revision requests as required, and the specific completion details are outlined below:

1. **Manuscript File Inventory Arrangement**

We have checked the submission file inventory in the Editorial Manager system and marked the **description column** of the most recent manuscript file with "latest" for clear identification, ensuring the editorial team can easily locate the valid version for review.

2. **Role of the Funders Statement**

The role of funder has been stated in the cover letter as instructed.

3. **Manuscript Title Consistency**

We have thoroughly verified the manuscript title and ensured the title displayed on the online submission form is **identical** to that in the manuscript document, with no discrepancies existing between the two.

4. **Author Addition**

We have updated the submission data via the "Edit Submission" function in the system and successfully added **Ivy Meihua Su** to the author list, with all relevant author information completed accurately.

Thank you again for your patience and support for our work. Should you need any further information or clarification, please do not hesitate to contact us at any time.

Sincerely,

Winsy Wing Sze Wong
