# Supplementary material for: Interactions in psychosocial interventions in dementia care: A systematic review protocol
Source: PLoS One. 2026 May 6;21(5):e0348775. doi: 10.1371/journal.pone.0348775 (PMC13148684; doi:10.1371/journal.pone.0348775)
Supplement: Attachment — Submitted filename: Response to Reviewers.docx [file pone.0348775.s005.docx]

Winsy Wing Sze Wong, Ph.D.
Corresponding Author
Department of Language Science and Technology
The Hong Kong Polytechnic University
Hung Hom, Kowloon, Hong Kong
Email: winsyws.wong@polyu.edu.hk
Phone: (852) 2766 7454
March 3, 2026

Sascha Köpke
Editorial Office
PLOS ONE

**Re: Response to Reviewers for Manuscript PONE-D-25-60484**

**Title: Interactions in psychosocial interventions in dementia care: A systematic review protocol**

Dear Dr. Köpke,

Thank you for the opportunity to revise our manuscript. We appreciate the thoughtful and constructive feedback provided by you and the reviewer. We have **fully and carefully addressed all revision requirements**—including the initial administrative submission requests, the academic methodological comments, the reviewer’s substantive suggestions, and the latest journal submission guidelines. All modifications have been clearly highlighted in the tracked version of the manuscript, and all authors have reviewed and approved the fully revised manuscript and all accompanying submission materials.

We confirm that all prior administrative revision requirements have been fully implemented and retained in this resubmission, and we have strictly complied with all new editorial instructions for the review process. Below is a comprehensive point-by-point response to all raised comments and revision requests.

**I. Initial Administrative Revision Requirements (Fully Implemented & Retained)**

1. **Manuscript File Inventory Arrangement**

We have checked the submission file inventory in the Editorial Manager system and marked the description column of the most recent manuscript file with "latest" for clear identification, ensuring the editorial team can easily locate the valid, up-to-date version for review; no outdated or absent files are present in the inventory.

2. **Role of the Funders Statement**

As instructed, the funder role statement has been clearly stated in the cover letter for this resubmission, and the updated funding disclosure has been revised in line with journal requirements (see Section IV for full statement). The core declaration remains unchanged: *The funders had no role in study design, data collection and analysis, decision to publish, or preparation of the manuscript.*

3. **Manuscript Title Consistency**

We have thoroughly verified and confirmed full consistency of the manuscript title (**Interactions in psychosocial interventions in dementia care: A systematic review protocol**) across the online submission form and the manuscript document, with no discrepancies existing between the two.

4. **Author Addition**

We have updated the submission data via the "Edit Submission" function in the Editorial Manager system and successfully added **Ivy Meihua Su** to the author list, with all relevant and complete author information accurately completed and retained in the system.

**II. Response to Academic Editor’s Comments**

1. **Clarification of Objectives (Relation between interactions and outcomes)** *Comment:* The abstract states the second objective aims at the “relation between interactions and outcomes,” which sounds like reporting actual results.

*Response:* We agree that the original wording was ambiguous. We have clarified in the Objective section that our aim is to *investigate whether and how* existing studies have examined this relationship, rather than conducting a meta-analysis of the relationship ourselves at this stage. We will synthesize the reported evidence on this relationship. 

*Action:* The objective text in the abstract has been edited ‘investigate whether existing studies have investigated the relationship between measures of interaction and treatment outcomes, if yes, further investigation will be carried out to describe the relationship between measures and outcomes’ (p.3, lines 28-33). And research objective 4 in the main text has been refined to: “further investigation will be carried out to describe the relationship between measures and outcomes” (p. 13, lines 189-191 in the manuscript).

2. **Campbell Registration and Journal Choice** *Comment:* Please provide a link to the registration. Why publish in PLOS ONE if it is a Campbell Review?

*Response:* We did not intend to submit the manuscript to Campbell review. We applied Campbell Review for a title registration and were accepted and listed on their website: <https://onlinelibrary.wiley.com/doi/10.1002/cl2.70067>

We have chosen to publish the protocol in PLOS ONE to reach a broader, interdisciplinary audience (psychology, nursing, linguistics) that extends beyond the traditional scope of Campbell, ensuring the methodology receives wide visibility.

3. **Participants and Cutoff** *Comment:* Is there a cutoff for mixed populations (e.g., 80%)?

*Response:* Yes, we have established a clear cutoff. *Action:* We have specified in the “Eligibility criteria" section: "Studies involving a mixed population will be considered only if 80% of the population consists of people living with dementia” (p. 14, lines 207-208 in the manuscript and item 8, Participants in the PRISMA list).

4. **Timeframe for Search** *Comment:* The timeframe should be updated; there is no reason to limit to April 2025.

*Response:* We apologize for the confusion caused by the future date in the previous draft.

*Action:* We have updated the search strategy to state that the search will be conducted “from inception to December 31, 2025” (p.4, line 39 in the manuscript and items 8, 9, and 10, appendix in PRISMA list). We removed the exclusion criterion regarding the future date.

5. **Critical Appraisal Instruments** *Comment:* Please select an appropriate instrument for each design and explain how quality assessment connects to results.

*Response:* We have specified the tools for each design: JBI Checklist for RCTs, JBI for Quasi-Experimental, JBI for Cross-Sectional, and the COSMIN Risk of Bias checklist for psychometric studies. We also clarified that quality assessment will inform the narrative synthesis (giving greater weight to studies with lower risk of bias).

*Action:* The "Critical Appraisal" section has been expanded with these specific details (p.18-19, lines 290-297-2 in the manuscript and item 14 in the PRISMA list).

6. **Screening Process Clarification** *Comment:* It is unclear if all full texts are reviewed by two independent reviewers. The PRISMA-P document states independent checking.

*Response:* This was a phrasing error in the previous draft. We confirm that **all** titles/abstracts and **all** full texts will be screened by two independent reviewers. The mention of "10%" referred to the initial pilot testing of the screening form to ensure inter-rater reliability, not the screening itself.

*Action:* We have rewritten the "Screening and inclusion" section to clearly state that two independent reviewers will screen all records at both stages (p.17, lines 264-269 in the manuscript).

7. **PRISMA-P Synchronization** *Comment:* The checklist contains steps not reported (e.g., SWiM, subgroup analysis).

*Response:* We have synchronized the text with the checklist. We are utilizing the SWiM (Synthesis Without Meta-analysis) guidelines for the narrative synthesis.

*Action:* We have explicitly cited the SWiM guidelines in the "Data Synthesis" section (p.19, lines 303-310 in the manuscript, and items 15c, 16, 17 in the PRISMA list).

8. **Language (Colloquialisms)** *Comment:* Avoid "till" and "myriad."

*Response:* We have removed these terms.

*Action:* "Till" has been replaced with "until" (or removed in the context of the date update), and "myriad" has been replaced with "variety" or "diverse" (p. 4, line 38; p.4, line 51 in the manuscript)

**III. Response to Reviewer #1’s Comments**

1. **Terminology (People living with dementia)** *Comment:* Please avoid abbreviations for people living with dementia.

*Action:* We have removed the abbreviation "PwD" and replaced it with "people living with dementia" throughout the manuscript (p. 3, line 21, 24; p. 4, line 39; p. 6, line 64; p.7, line 73, 85, 90; p. 8, line 101, 109; p. 9, line 111, 114, 119, 120, 125, 129; p. 10, line 103, 135, 139, 141, 144; p. 11, line 151, 154, 156, 161, 163, 164, 167; p. 12, line 169, 171, 183; p. 13, line 194; p. 14, line 211; p. 15, line 231, 234; p. 21, line 332, 333, 334 in the manuscript).

2. **Terminology (Elderly)** *Comment:* Please avoid the word "elderly."

*Action:* We have replaced "elderly" with "older adults" or "older people" throughout the text, consistent with the Journal of Geriatric Physical Therapy recommendations.

3. **Abstract (Intervention Definition)** *Comment:* Definition of non-pharmacological interventions seems to mean psychosocial interventions.

*Action:* We have updated the title and the text to specifically refer to "psychosocial interventions" to be more precise and aligned with our inclusion criteria (p. 3, lines 20-26, lines 29; p. 4, line 40, 51; p. 7, lines 80-82, line 86, 91; p. 8, line 103, 105; p. 9, line 129; p. 11, line 166; p. 12, line 168, 175, 178; p. 13, line 193; p. 14, line 211, 215-216; p. 15, line 232, 234; p. 21, line 332, line 333, line 337 in the manuscript).

4. **Introduction (Lancet/Statistics)** *Comment:* Reference the Lancet paper regarding risk factors and prevention. Point out declining age-related incidence vs. rising absolute numbers.

*Action:* We have incorporated the *Lancet Commission (2020)* reference and added a sentence in the Introduction highlighting that while age-specific incidence may be declining in some Western countries, the absolute number of cases continues to rise, emphasizing the need for intervention (p. 6, lines 60-72 in the manuscript).

5. **Review Type (Systematic vs. Scoping)** *Comment:* Consider if this is a systematic or scoping review.

*Response:* We would like to clarify that this is a systematic review because we aim to critically appraise the quality of the studies (using JBI/COSMIN tools) and synthesize evidence on the measurement properties and outcomes, rather than just mapping key concepts.

*Action:* We have maintained the systematic review designation but ensured our methodology (critical appraisal and synthesis) supports this (p. 3, line 28; p.12, line 180; p. 13, line 196; p. 14, lines 221-223; p. 19, lines 303-310 in the manuscript and item 8, study design in the PRISMA list).

6. **Inclusion of FTD** *Comment:* Why didn’t you consider people with FTD?

*Response:* We do include FTD.

*Action:* In the "Types of participants" section, we have explicitly listed "primary progressive aphasia" (a form of FTD) and "dementia of any type," ensuring FTD is covered. Also in the search terms (provided in the Appendix 1 of PRISMA-P checklist), frontotemporal dementia is added (p. 8 of PRISMA checklist).

7. **Grey Literature and Citation Tracking** *Comment:* How do you search grey literature and conduct citation tracking?

*Action:* We have added details to the "Searching other sources" section, specifying that we will search ProQuest Dissertations and Theses, Google Scholar, and conference proceedings. We also clarified that we will perform backward citation tracking on included studies (p. 16, lines 246-259 in the manuscript and items 9 and 10 in the PRISMA list).

8. **Search Date (April 2025)** *Comment:* Rephrase the sentence about studies published after April 2025.

*Action:* As noted in the response to the Editor, we have removed the future cutoff date and stated the search covers "inception to December 31, 2025" (p.4, line 38-39; p.15, line 236 in the manuscript)

9. **Screening Procedure** *Comment:* "Entries will be divided into two parts" is misleading.

*Response:* We agree.

*Action:* We deleted the confusing sentence. The text now clearly states that two independent reviewers (Su and Li) will screen the titles/abstracts of *all* identified records (p. 17, lines 264-269 in the manuscript).

10. **Third Reviewer** *Comment:* Don’t understand why a third reviewer is reviewing 10% of all titles.

*Response:* This was a remnant of a previous draft regarding reliability checks.

*Action:* We have clarified that the third reviewer (Author Wong) serves as an arbitrator to resolve disagreements between the two primary reviewers (p. 17, lines 268-269 in the manuscript).

11. **Library Consultation** *Comment:* Was a library included in the development of the search string?

*Response:* The search strategy was developed by the research team using standard MeSH terms and keywords derived from key literature in the field. We also consulted the resources, guides, and tutorials provided by the library of our institute. (<https://libguides.lb.polyu.edu.hk/syst_review/find_SR>)

**IV. Compliance with PLOS ONE Journal Submission & Style Requirements**

1. **Required Submission Files**

In accordance with your latest editorial instructions, we have prepared and uploaded **all required supplementary files** as separate documents in the Editorial Manager’s "Submissions Needing Revision" folder, labeled exactly as specified:

- *Response to Reviewers*: This comprehensive document with a detailed point-by-point response to all Academic Editor and Reviewer #1 comments;
- *Revised Manuscript with Track Changes*: A marked-up copy of the manuscript clearly highlighting all modifications made in response to the review process;
- *Manuscript*: A clean, unmarked version of the revised manuscript with no track changes or editorial marks.

2. **PLOS ONE Style Requirements**

The manuscript fully adheres to all PLOS ONE style guidelines, including correct file naming conventions, template formatting, and editorial writing standards.

3. **Funding Statement and Acknowledgments**

Per journal request, all funding-related text has been removed from the Acknowledgments section of the manuscript. The **updated Funding Statement** (to be reflected in the online submission system) is:

*This protocol is supported by the Health and Research Medical Fund of the Health Bureau of the Government of the Hong Kong Special Administrative Region of the People’s Republic of China (reference number: 22231471) and the General Research Fund of the Research Grants Council of the Hong Kong Special Administrative Region of the People’s Republic of China (reference number: 15602024). The funders had no role in study design, data collection and analysis, decision to publish, or preparation of the manuscript.*

4. **Data Availability**

We confirm that all data underlying the findings of this systematic review will be made fully available without restriction at the time of publication. As this is a systematic review protocol, no research datasets have been generated to date.

5. **Funder Role & Financial Disclosure**

No changes have been made to our financial disclosure, and the core funder role statement remains consistent in the cover letter and manuscript: *The funders had no role in study design, data collection and analysis, decision to publish, or preparation of the manuscript.*

We believe the manuscript is now significantly improved and ready for publication.

Sincerely,

Winsy Wing Sze Wong Department of Language Science and Technology The Hong Kong Polytechnic University
